# Propylthiouracil-induced lupus-like or vasculitis syndrome

**DOI:** 10.1186/2049-6958-7-14

**Published:** 2012-07-17

**Authors:** Cuneyt Tetikkurt, Mehmet Yuruyen, Seza Tetikkurt, Nihal Bayar, Imran Ozdemir

**Affiliations:** 1 Pulmonary Diseases Department, Cerrahpasa Medical Faculty, Istanbul University, Tanzimat sokak Serkan Apt. No 8/16 Caddebostan, Istanbul, 34728, Turkey; 2 Internal Medicine Department, Cerrahpasa Medical Faculty, Istanbul University, Istanbul, Turkey; 3 Pathology Department, Bagcılar Training and Research Hospital, Istanbul, Turkey

**Keywords:** Lupus-like syndrome, Propylthiouracil, Vasculitis

## Abstract

A 27 year old female with Graves’ disease presented with fever, exertional dyspnea and polyarthralgia. Erythema nodosum had occured three months earlier. The patient declared irregular use of propylthiouracil (PTU) for the last 8 months. Neutropenia and microscopic hematuria developed in the second week of admission. Chest X-ray showed inhomogenous pulmonary opacities, left pleural effusion and cardiomegaly. Computed tomography (CT) revealed multiple subpleural nodules, left pleural effusion, pericardial effusion, enlarged mediastinal and axillary lymph nodes. Bronchoalveolar lavage (BAL) cytology demonstrated hemosiderin laden macrophages. Histopathologic examination of the transbronchial biopsy specimen revealed a nonspecific inflammation. Serum was positive for ANA, P-ANCA, MPO-ANCA, PR3-ANCA and negative for anti-ds-DNA, C-ANCA, C3, C4 and anti-histone antibody. All symptoms resolved in two months after PTU withdrawal and starting steroid treatment. The same clinical manifestations recurred when the patient used PTU erronously one month after discharge.

This is a case of PTU induced-autoimmune disease in whom the accurate distinction between drug-induced-lupus (DIL) and vasculitis was not possible due to the significant overlap of clinical and laboratory findings causing a significant diagnostic challenge for the chest physician.

## Background

PTU can cause adverse reactions including fever, rash, leucopenia, arthiritis, vasculitis and lupus-like syndrome [1]. However, pulmonary complications like interstitial pneumonia, adult respiratory distress-like syndrome and pleural effusion are extremely rare [2,3] The mortality rate is low and related to systemic manifestations. PTU-induced lupus is now well established and many other autoimmune disorders including vasculitis have been reported. Most of the cases of PTU-induced autoimmune phenomena are due to vasculitis.

We present the case of a patient who developed erythema nodosum, pericardial and pleural effusion, subpleural nodules, microhematuria, alveolar hemorrhage, mediastinal and axillary lymph node enlargement due to PTU treatment. Although there are significant differences in the clinical features of drug-induced lupus (DIL) and vasculitis that allow an accurate diagnosis, the distinction was not possible in our case because of substantial overlap of clinical findings.

## Case presentation

A 27 year old woman with a history of Graves’ disease was admitted for fever, exertional dyspnea and polyarthralgia. She had started on PTU (150 mg p.oqd) for Graves’ disease which she had used irregularly during the last 8 months. There was no other past medical history of interest.

**Figure 1:**
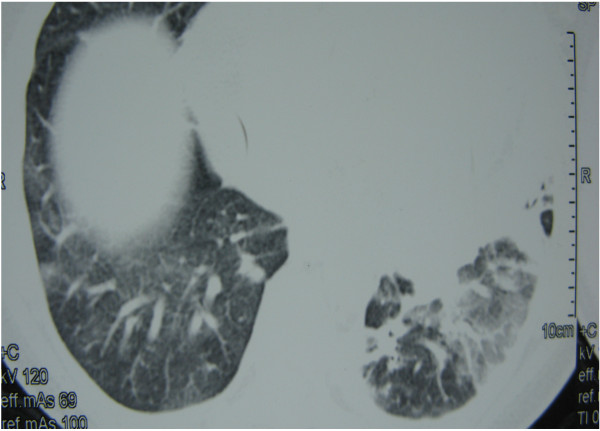
Chest CT showing bilateral subpleural nodules and left basal ground glass pattern.

Four weeks before admission the patient developed low grade fever (37.6°C), dry cough, exertional dyspnea, polyarthralgia and left sided pleural pain. Erythema nodosum had occured three months earlier. WBC was 3.26 × 10^3^/ml with 59 percent neutrophils, CRP: 155, ERS: 74 mm/h. Blood biochemistry was within normal limits. Urine analysis revealed 10 to 12 red cells per low power field and mild proteinuria. ECG revealed sinus tachycardia (112/min). There was no radiologic abnormalitiy on joint films. Chest x-ray showed cardiomegaly, left pleural effusion and bilateral inhomogenous opacities. Tuberculin test was negative. Arterial blood gases; pH: 7.44, pO_2_: 76.2, and pCO_2_: 36.8 mm Hg. Thyroid function tests were normal. Chest CT showed pericardial and left pleural effusion, bilateral subpleural nodules, left basal ground glass pattern (Figure 1), multiple mediastinal and bilateral axillary lymph nodes. Serum D-dimer was 4.57 mg/L. Doppler ultrasonography of the legs was normal. She was given a course of antibiotics for seven days with no improvement of her symptoms.

**Figure 2:**
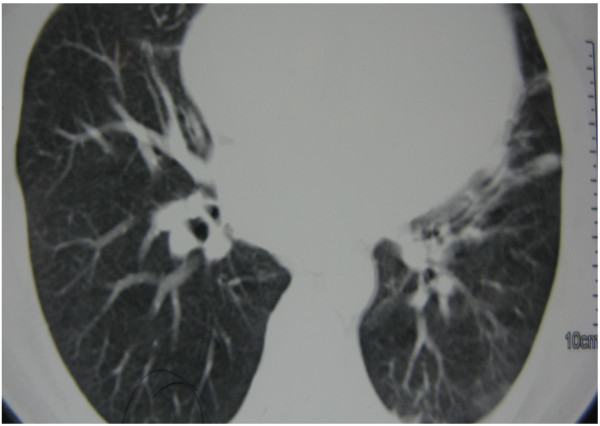
Resolution of radiologic findings after PTU withdrawal.

Serologic tests, including rheumatoid factor, anti-double-stranded deoxyribonucleic acid, complement levels, antiglomerular basal membrane antibody, and Brucella agglutination test were negative. Serum antinuclear antibodies (ANA: +, homogeneous), perinuclear anti-neutrophil cytoplasmic antibodies (P-ANCA: +), cytoplasmic anti-neutrophil cytoplasmic antibodies (C-ANCA: +), antimyeloperoxidase antibodies (MPO-ANCA: 135.4 EU/ml), and antiproteinase 3 antibodies (PR3-ANCA: 27.8 EU/ml) were positive. The level of anti-thyroperoxidase antibodies (anti-TPO) was high (242.96 IU/ml). Pleural fluid was an exudate with lymphocyte predominance (68%). The culture and the cytologic examination of the pleural fluid was not diagnostic. BAL showed hemosiderin-laden macrophages with a normal differential cell count. Culture of the BAL fluid was negative for bacteria, tuberculosis and fungus. Histopathologic examination of the transbronchial specimen did not demonstrate any features of vasculitis, granulomatous changes or fibrosis but only nonspecific inflammation. Serum anti-histone antibody was negative. PTU treatment was stopped. Methamizole (30 mg/day) and methylprednisolone (40 mg/day) were commenced. Symptoms, laboratory, and radiologic findings completely resolved within two months of PTU withdrawal (Figure 2). Identical clinical manifestations, including the laboratory findings, recurred when the patient erroneously used PTU again instead of methamizole for six weeks after the hospital discharge, when the resolution of symptoms had been reached in four weeks after the discontinuation of the drug. The patient had an uneventful recovery following thyroidectomy.

## Discussion

It has long been known that PTU can induce an adverse autoimmune response with DIL or vasculitis [4]. Cases can be classified into DIL or vasculitis according to the accepted definitions, clinical and serological features. Here we describe the case of a patient who presented with a clinical picture of vasculitis during PTU treatment for Graves’ disease. Our patient had both findings of DIL, in relation to the regular long-term medication use, and vasculitis [5]. After PTU withdrawal, an excellent outcome took place with resolution of all symptoms, clinical, laboratory, and radiologic findings, however the same clinical picture occurred again when the patient inadvertently used PTU again. The patient was a diagnostic dilemma for the clinician because the risk of developing DIL with using PTU is less than 1% of the treated patients [6]. Moreover the distinction between DIL and ANCA-associated vasculitis was not possible because of the significant overlap between these two entities in our case. Finally the patient did not fit into any collagen disease profile.

Aloush and coworkers have provided clear distinction between the DIL and ANCA-associated vasculitis reviewing their similarities and differences. Patients with PTU-induced DIL have more musculoskeletal complaints, more serositis and gastrointestinal involvement. On the contrary, the ANCA-associated vasculitis more frequently presents upper airways, pulmonary and renal involvement. ANA, anti-DNA, and anti-histone antibodies are predominantly found in DIL, whereas p-ANCA are found in a similar proportion of patients in both groups. c-ANCA are detected only in patients with vasculitis. All DIL patients recovered completely after withdrawal of PTU, while about 50% of PTU-induced vasculitis need steroids or immunosuppressive drugs [7]. Nearly every feature of both entities was identified in our patient with a significant overlap of serologic markers. DIL includes various manifestations like arthralgia, pericarditis, pleuritis, and fever, which were all present in this patient. Antinuclear antibodies are positive in over 50% of cases and native anti-DNA are reported [8]. These markers were negative in our case. Resolution of symptoms and radiologic findings after drug withdrawal suggested strongly the DIL associated with PTU treatment, while negative serum anti-histone antibody with positive C-ANCA, and the need for steroid immunosupression indicated PTU-induced-vasculitis.

Some patients receiving PTU develop a different and a more serious kind of adverse autoimmune response characterized by high titres of antibodies to MPO with clinical manifestations including pauci-immune necrotizing and crescentic glomeruloneprithis, upper respiratory tract disease, and pulmonary hemorrhage with negative or low titres of ANA [9,10]. Our patient had microhematuria and alveolar hemorrhage that were diagnosed during the clinical investigation of a possible autoimmune disorder. Detection of MPO-ANCA with alveolar hemorrhage and microhematuria supports the presence of alveolar and glomerular vasculitis as Bosch has suggested [11].

PTU-induced vasculitis often occurs in a few weeks of treatment but may develop after months or years. The disorder is not usually dose dependent and may improve after discontinuation of the drug [12]. The patient was not compliant and declared very irregular use of PTU in the previous 8 months when the use of PTU had started. Antibodies to double-stranded DNA were absent and complement levels were normal. The high levels of PR3-ANCA and MPO-ANCA, which is thought to be a specific marker of anti-thyroid drug-induced vasculitis, decreased in correlation with the course of symptoms and the resolution of radiologic findings after PTU withdrawal.

The mechanism responsible for ANCA and vasculitis in patients on PTU is not well understood. ANCA production may occur as a result of the interaction between PTU and neutrophils or neutrophil MPO. MPO and hydrogen peroxide produced by neutrophils can metabolize the drug leading to the reactive intermediates that are immunogenic for T-cells and stimulate the immune system [12,13]. The metabolites which have a cytotoxic activity determine the cell death and production of autoantibodies [14]. Because human MPO and thyroid peroxidase (TPO) are the members of the same gene family, patients with thyroid disease and TPO antibodies may develop cross-reactivity to MPO. TPO and MPO antibodies are often both present in these patients [15] as may be the case for our patient. Serious adverse effects required corticosteroid treatment. Complete recovery of clinical and laboratory findings occured following the cessation of PTU and starting of the steroid treatment. The same clinical picture recurred after the erroneous use of PTU by the patient.

Regardless of the mechanism, the association between PTU treatment and ANCA-associated vasculitis or DIL is rare but well described. Substantial differences in clinical, serologic and outcome of these syndromes provide an accurate diagnosis [7]. The systemic adverse effects of PTU may also be difficult to distinguish from the manifestations of Graves’ disease or from other vasculitides. Antineutrophil cytoplasmic antibodies and especially high levels of anti-histone antibodies are useful for the diagnosis of PTU associated lupus-like syndrome. It is important to be aware of this complication because early withdrawal of the drug results in clinical recovery and may prevent a fatal outcome.

Our patient had severe respiratory problems with erythema nodosum as the initial sign. Neither the clinical manifestations and nor the serologic markers of our patient indicated DIL, ANCA-associated vasculitis or collagen disease accurately. Presence and resolution of enlarged mediastinal and axillary lymph nodes after drug withdrawal would also strongly suggest drug induced autoimmune disease in this case. Different presentations, overlap syndromes associated with PTU-induced disease, various clinical and laboratory findings may represent a diagnostic challenge for the pulmonary clinician. The current case did not fit into any of the two previously described classifications.

## Conclusions

In addition to the negligible incidence of PTU-induced disease, the overlapping symptoms and laboratory findings may lead to a diagnostic conflict. Consequently, putting such patients into the PTU-induced autoimmune disease classification as opposed to the DIL or ANCA-associated vasculitis would better describe the clinical features.

## Consent

Written informed consent was obtained from the patient for publication of this case report and any accompanying images. A copy of the written consent is available for review by the Editor-in-Chief of this journal.

## Competing interests

The authors declare that they have no competing interests.
